# Reliability of a 1-week recall period for the Medical Outcomes Study Sleep Scale (MOS-SS) in patients with fibromyalgia

**DOI:** 10.1186/1477-7525-7-12

**Published:** 2009-02-10

**Authors:** Alesia Sadosky, Ellen Dukes, Chris Evans

**Affiliations:** 1 Pfizer Global Outcomes Research, 235 East 42nd Street, New York, NY 10017, USA; 2 Mapi Values, 15 Court Square, Suite 620, Boston, MA 02108, USA

## Abstract

**Objective:**

To evaluate the reliability of a one-week versus a four-week recall period of the Medical Outcomes Study Sleep Scale (MOS-SS) in patients with fibromyalgia (FM).

**Methods:**

The MOS-SS was administered by mail to patients with a confirmed diagnosis of FM and a current pain rating of > 2 (0–10 point numerical rating scale) recruited through newspapers, support groups, and the Internet. Reliability of MOS-SS subscale domains was evaluated using test-retest methodology separated by a 1–3 day interval for the 4-week recall period and a 7-day interval for the 1-week recall period. Patient Impression of Change was evaluated for sleep, and for patients with no change, the intraclass correlation coefficient (ICC) and the Pearson correlation coefficient was calculated for MOS-SS subscales.

**Results:**

Of 129 patients enrolled, 91.3% were female, mean age was 49.4 ± 11.0 years; self-rated FM severity was moderate-to-severe in 88.1% of patients. MOS-SS subscale scores were similar for both recall periods with little variation between test-retest. The 9-item Sleep Problems Index scores ranged from 57.2 ± 14.5 to 61.9 ± 15.8 across all assessments and demonstrated high reliability which was similar for the 1-week (ICC 0.81) and 4-week (ICC 0.89) recall periods. For the other MOS-SS subscales, the 1-week recall period also showed good reliability, which was consistent for the ICC and Pearson correlation coefficients.

**Conclusion:**

A 1-week recall period is adequately reliable for use of the MOS-SS in studies evaluating sleep disturbance in patients with FM.

## 

Although the etiology of fibromyalgia (FM) is uncertain, it is generally diagnosed according to the American College of Rheumatology (ACR) criteria, which include chronic, widespread pain for at least 3 months, and the presence of 11 out of 18 tender points [1]. It has been estimated that approximately 5 million individuals in the U.S. have FM, with a higher prevalence among women (3.4%) than men (0.5%) [2]. In other countries, the prevalence of FM has been estimated to range between 0.5% to 5%, also with a higher prevalence among women [3].

FM is associated with a substantial socioeconomic burden resulting from excess health resource utilization, reductions in productivity and quality of life, and a health status that is poorer than other chronic pain conditions such as rheumatoid arthritis and osteoarthritis [4-7]. This burden is derived not only from the pain, which is characteristic of FM and is considered the hallmark symptom, but also from a constellation of other symptoms including sleep disturbance, which after pain, is a major complaint of patients with FM and may be an integral component of the condition. Specific alterations in sleep architecture have been documented in patients with FM by polysomnography [8-10], suggesting an association between sleep dynamics and the underlying pathophysiology.

There is an overall reciprocal relationship between sleep disturbance and pain [11-13], with correlation between these outcomes reported in several rheumatologic conditions including FM [14]. A recent study in patients with FM suggests sleep disturbance may be predictive of pain [15]. Not surprisingly, both pain and sleep are considered core domains essential for evaluation in FM clinical trials [16].

A variety of sleep instruments are available for evaluating sleep disturbance and its impact [17], including a new scale for evaluating restorative sleep (Sleep Quality Assessment; SQA) [18]. A review of sleep assessment instruments for use in chronic pain clinical trials suggested that while none of the currently available instruments are optimal, the Medical Outcomes Study Sleep Scale (MOS-SS) [19] may represent the best choice [20]. This recommendation was based on overlap between key sleep constructs that should be evaluated with the domains that are assessed by the MOS-SS.

The psychometric properties of the MOS-SS have been evaluated in patients with a variety of conditions characterized by pain including neuropathic pain [21,22], restless legs syndrome [23], and fibromyalgia [24]. These studies consistently demonstrated its validity and reliability for assessing the key constructs of sleep quality and quantity, and that it is also sensitive to change, suggesting its utility in clinical trials. However, it has a recall period of 4 weeks and responses may consequently be subject to recall bias, potentially compromising the accuracy of assessment. Such recall bias provides the basis for recommendations by the FDA against the use of patient-reported outcomes with long recall periods [25]. Therefore, the purpose of this study was to evaluate the test-retest reliability of a one-week recall period of the MOS-SS compared with the four-week recall period in patients with FM.

## Methods

The MOS-SS was included in a stand-alone, longitudinal study conducted between May and September 2007 designed to evaluate the psychometric properties of several outcomes assessment instruments in patients with FM. Patients were recruited through newspapers, support groups, and the Internet, and were compensated for participation. Individuals were included if they were ≥ 18 years old and provided a confirmed physician diagnosis of FM for at least 3 months prior to enrollment; the participants' clinicians forwarded written confirmation of the diagnosis of FM to the study investigators. Individuals were required to have a current pain rating of > 2 on an 11-point numerical rating scale (NRS) to enable enrollment of individuals with a broad range of pain severity, since another outcome of this study was validation of cutpoints representing moderate and severe pain as previously described [26]. It is also likely that use of this rating resulted in a more representative population than limited by the score ≥ 4 generally required for inclusion in clinical trials of FM [27-33]. The ability to read/understand English and cooperate with investigators and study procedures were also required. Exclusion criteria included a previous diagnosis of rheumatoid arthritis or systemic lupus erythematosus and/or any other chronic painful condition that could confound the ability to distinguish other chronic pain from pain related to FM. Eligible participants completed informed consent and study participation forms; protocol and study documents were approved by the appropriate Institutional Review Board.

The MOS-SS is a 12-item questionnaire that aims to evaluate key constructs of sleep, with derived subscales for the domains of sleep disturbance (4 items), quantity of sleep (1 item), snoring (1 item), awakening due to short of breath or with headache (1 item), sleep adequacy (2 items), and somnolence (3 items) [19]. Additionally, a 9-item Sleep Problems Index can be generated which assesses overall sleep problems. It includes the 4 sleep disturbance and the 2 sleep adequacy items, 2 of the somnolence items, and awakening short of breath/headache; higher scores indicate greater sleep impairment, and this index is often used in clinical trials as an indication of sleep quality.

The MOS-SS was administered as a mailed questionnaire. All patients completed both the 4-week and 1-week recall period versions of the MOS-SS, with the 4-week recall period questionnaires administered first. The test and retest of the MOS-SS using the 4-week recall period were separated by an interval that ranged between 1 and 3 days, and for the 1-week recall period, the test and retest were separated by a 7-day interval. At the time of the retest, patients also evaluated their impression of change in sleep (Patient Impression of Change; PIC) for the period between the test and retest. The PIC was adapted from the Patient Global Impression of Change for the purpose of this study by specifying a change in sleep due to FM, but was based upon methodology widely used to assess degrees of change [34]. As with the PGIC, it is answered on a 7-point scale of 1 = very much improved; 2 = much improved; 3 = minimally improved; 4 = no change; 5 = minimally worse; 6 = much worse; 7 = very much worse.

Paired t-tests were used to determine significance of the difference between test and retest scores. To evaluate test-retest reliability of the subscales, the intraclass correlation coefficient (ICC) using Shrout-Fleiss reliability (single-score statistic) was calculated from paired values for each recall period [35]. A value greater than the conventionally accepted threshold of 0.70 was considered an indication of reliability [36]. Pearson correlation coefficients were also calculated as confirmation of the reliability estimates. This assessment for reliability was performed on data from stable subjects with respect to the PIC, i.e. patients who reported "no change."

## Results

A total of 129 patients with FM were enrolled; 91.3% were female, and the mean age was 49.4 ± 11.0 years. Self-rated FM severity was at least moderate in 88.1% of patients, and 88.3% reported a duration of FM of at least 2 years since diagnosis. Approximately two-thirds of the patients (68.3%) reported taking medications for their FM.

**Figure 1:**
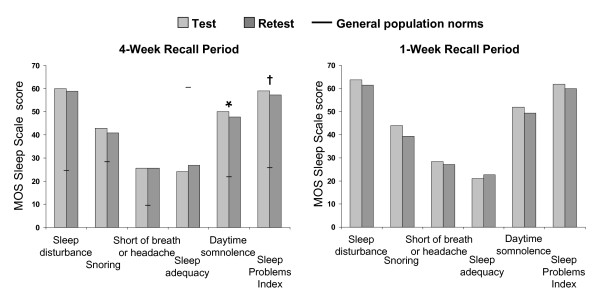
**Test and retest scores on the MOS-Sleep Scale for the 4-week and 1-week recall periods**. General population norms for the U.S. population (not adjusted for age and gender), shown as black horizontal bars, are provided for reference from Hays et al. [21] only for the 4-week recall period, since population norms have not been established for a 1-week recall period. * p = 0.0062 and † p = 0.01 for the difference between test and retest using paired t-tests.

The mean test and retest scores for the MOS-SS domains and the 9-item Sleep Problem Index, along with general US population norms that were derived in a validation study using the 4-week recall period [21], are shown in Figure 1. Scores were similar using the 4-week and 1-week recall periods, and generally showed little variation between the test and retest. The only significant differences between test and retest values were observed for the domains of Daytime Somnolence (p = 0.0062) and the 9-item Sleep Problems Index (p = 0.01) for the 4-week recall period.

All domain scores for the 4-week recall period showed substantial differences from population norms, indicating the presence of sleep impairment. Sleep Disturbance and Daytime Somnolence were the MOS-SS domains which had the greatest negative impact on sleep quality for both the 4-week and 1-week recall period. The Sleep Problems Index scores, which are shown in Table 1, ranged from 57.2 ± 14.5 to 61.9 ± 15.8 across all assessments, and were more than twice as high as reported for the general population norm (25.8) [21], demonstrating substantial sleep problems in these patients.

**Table 1:** Test and retest scores for the 9-item Sleep Problems Index of the Medical Outcomes Study Sleep Scale.

**Recall period**	**Test (n)**	**Retest (n)**	**Change (n)**	**p**
4 weeks	59.01 ± 14.85 (79)	57.23 ± 14.46 (79)	-2.0 ± 6.7 (78)	0.01
1 week	61.9 ± 15.8 (62)	59.9 ± 15.2 (62)	-2.0 ± 9.5 (62)	0.11

Although 60.4% and 56.9% of patients reported no change in sleep status on the PIC between the test and retest for the 4-week and 1-week recall periods, respectively, improvement and worsening were both reported by patients. For the 4-week recall period, 8.2% of patients reported improvement and 23.8% reported worse sleep. Similarly, for the 1-week recall period, 16.5% reported improved sleep and 26.7% reported worse sleep.

The test-retest reliability of the MOS-SS domains and the 9-item Sleep Problems Index was assessed for the 78 patients (60.4%) and 62 patients (48.1%) who reported no change on the PIC for the 4-week and 1-week recall periods, respectively. The ICC and Pearson correlations for these patients are shown in Table 2. Except for the Sleep Adequacy domain, the ICC required threshold of 0.70 was exceeded for both recall periods with slightly higher values for the 4-week period. For the Sleep Adequacy domain, although the ICC did not achieve the threshold value, the 1-week recall period resulted in a higher value (0.69) than the 4-week recall period (0.63). Values of the Pearson correlation coefficient were comparable to the ICC.

**Table 2:** Test-retest reliability of the Medical Outcomes Study Sleep Scale. Results are for stable subjects, defined as patients who report 'No Change' on Patient Impression of Change sleep question, with non-missing values for both test and retest.

**Domain**	**4-Week Recall**				**1-Week Recall**			
	
	n	Test-retest p value	Intra-class correlation^a^	Pearson correlation	n	Test-retest p value	Intra-class correlation^a^	Pearson correlation
Sleep disturbance	78	0.1707	0.93	0.93	62	0.2354	0.76	0.76
Snoring	76	0.4385	0.90	0.90	61	0.0800	0.83	0.83
Awakening short of breath or with headache	78	0.7412	0.87	0.87	62	0.6154	0.73	0.73
Sleep adequacy	78	0.2094	0.63	0.64	62	0.3619	0.69	0.69
Daytime somnolence	78	0.0062	0.88	0.89	62	0.1222	0.84	0.84
9-Item Sleep Problems Index	78	0.0100	0.89	0.90	62	0.1088	0.81	0.81

^a ^Calculated using Shrout-Fleiss reliability: single score statistic.

## Discussion

The MOS-SS is a validated instrument for evaluating the impact of disease on sleep [19,21]. Its utility has been further demonstrated in chronic pain conditions by characterization of clinically important differences in patients with neuropathic pain [22] and FM [24]. In an effort to conform to recent recommendations for the use of patient-reported instruments with short recall periods [25], we evaluated the reliability of a 1-week recall period. The data reported here indicate that the MOS-SS produces comparable results regardless of the use of a 1-week or 4-week recall period. All domains and the 9-item Sleep Problems Index demonstrated adequate reliability that was similar for both recall periods when no change occurred in the underlying concept (e.g., sleep interference). Similar values suggesting high correlation were obtained for ICC and Pearson coefficients; ICC is considered a more conservative estimate of association than Pearson.

A limitation of this study is that these reliability estimates were based on patients who showed no change in sleep status between the test and retest. While the MOS-SS in general is sensitive to treatment effects, further evaluation of the 1-week recall period may be required under conditions characterized by a change in sleep disturbance (clinical trials and clinical practice). However, it should be noted that the psychometric evaluation of the MOS-SS in patients with FM utilized data from two clinical trials, one with a 4-week recall, and the other with a 1-week recall; both recall periods showed similar psychometric characteristics and sensitivity to change [24].

The fact that patients were compensated for participation is another limitation which may have introduced bias, since it is not known what effect the compensation may have had on the selection of patients for this study.

## Conclusion

The previously demonstrated psychometric soundness of the MOS Sleep Scale subscales and overall Sleep Problems Index combined with the current demonstration of the reliability of a 1-week recall period suggests the appropriateness of this instrument in the evaluation of sleep disturbance in patients with FM. However, further corroboration of the reliability of a 1-week recall period in clinical trials in patients with FM may be warranted.

## Abbreviations

FM: fibromyalgia; MOS-SS: Medical Outcomes Study Sleep scale; ICC: intraclass correlation coefficient; PIC: Patient Impression of Change

## Competing interests

Alesia Sadosky and Ellen Dukes are employees of Pfizer, Inc.; Chris Evans is an employee of Mapi Values, an outcomes research consulting company, which received funding from Pfizer to perform the analysis.

## Authors' contributions

All authors jointly contributed to the design of the study, data analysis and interpretation of results, and development of the manuscript. All authors have read and approved the content of the final manuscript.
